# New Anæsthetic

**Published:** 1867-09

**Authors:** 


### *New Anæsthetic.*

"We are glad to announce the introduction of a new anæsthetic, which, if further experience confirms the results hitherto obtained, promises to be of remarkable value. Dr. Protheroe Smith has been making some observations on the administration by inhalation of the tetrachloride of carbon ($CCl_4$), of which we wait for a fuller account. In the mean time, from our own observation, we may state in favor of this agent, that it has a pleasant odor, somewhat resembling that of the quince. We understand that anæsthesia is rapidly produced by it (in some cases in the space of half a minute,) that the condition appears to be easily sustained with or without entire loss of consciousness, and that the effects pass off very quickly. There is not usually, we learn, any excitement or struggling before anæsthesia supervenes, and its use is not followed by the sickness which is sometimes so troublesome a feature from the administration of chloroform. A point of great interest in relation to the tetrachloride of carbon is the property which we are told it possesses of immediately allaying pain arising from any cause. In a large number of instances it has been successfully employed for the relief of headache and dysmenorrhœal suffering. Dr. Protheroe Smith has found it of great value in inducing quiet and refreshing sleep. He has also employed it in midwifery, and finds that it removes pain without necessarily destroying consciousness, or interfering apparently with the expulsive efforts of labor."—*London Lancet.*

